# Additional statistical and graphical methods for analyzing site formation processes using artifact orientations

**DOI:** 10.1371/journal.pone.0190195

**Published:** 2018-01-02

**Authors:** Shannon P. McPherron

**Affiliations:** Department of Human Evolution, DeutscherPlatz 6, Leipzig, Germany, 04103; New York State Museum, UNITED STATES

## Abstract

The 3D orientation of clasts within a deposit are known to be informative on processes that formed that deposit. In archaeological sites, a portion of the clasts in the deposit are introduced by non-geological processes, and these are typically systematically recorded in archaeological excavations with total stations. By recording a second point on elongated clasts it is possible to quickly and precisely capture their orientation. The statistical and graphical techniques for analyzing these data are well published, and there is a growing set of actualistic and archaeological comparative data to help with the interpretation of the documented patterns. This paper advances this area of research in presenting methods to address some shortcomings in current methodologies. First, a method for calculating confidence intervals on orientation statistics is presented to help address the question of how many objects are needed to assess the formation of a deposit based on orientations. Second, a method for assessing the probability that two assemblages have different orientations is presented based on permutations testing. This method differs from existing ones in that it considers three-dimensional orientations rather than working separately with the two-dimensional bearing and plunge components. Third, a method is presented to examine spatial variability in orientations based on a moving windows approach. The raw data plus the R code to build this document and to implement these methods plus those already described by McPherron are included to help further their use in assessing archaeological site formation processes.

## Introduction

The 3D orientation of clasts within a deposit are known to be informative on processes that formed that deposit. In archaeological sites, a portion of the clasts in the deposit are introduced by non-geological processes, meaning typically through human behaviors but also in some instances from other biological agents such as carnivores. The find location of these non-geogenic clasts are typically systematically recorded in archaeological excavations, and with the introduction of total stations for precise piece proveniencing it became possible to record an additional point for elongated finds to quickly and precisely capture their orientation in the normal course of documenting the excavation [1, 2, 3]. Thus sometimes relatively substantial and informative data sets for the study of post-depositional formation processes can be amassed with little additional effort. The statistical and graphical techniques for analyzing these data are well published [1, 4, 5], and there is a growing set of actualistic and archaeological comparative data to help with the interpretation of the documented patterns [3, 6–26].

This paper advances this area of research in presenting several new methodologies for analyzing orientation data. These methods are designed to address several shortcomings in current methodologies. First, one difficulty in orientations analysis is in knowing how large a sample is needed to have a reliable or, more importantly, informative measure. Neudorf et al. [27] use eigenstatistics (see below) to argue that after approximately 35 measurements variability is minimized enough to achieve robust results. Lenoble and Bertran [5] use three data sets with unknown orientation characteristics to show that the Vector Magnitude (L) statistic (a measure of the strength of the dominant bearing in an assemblage) stabilizes after about 40 or 50 artifacts, and they argue that with this size of sample highly linear assemblages can be distinguished from other kinds of assemblages. In other words, the required sample size is in part a function of the distribution of orientations in the assemblage. This point is emphasized by Ringrose and Benn [28, 29] who also argue that 50 observations are required to reach a conclusion about the orientations. To show this and to provide a solution to the sample size problem, they propose a method for calculating confidence intervals for Benn diagrams [4]. While Benn diagrams have proven particularly useful for representing and comparing three dimensional orientations [1, 5], in a Benn diagram assemblages are represented by a single point with no indication of the associated uncertainty [28]. Thus to address these issues here, rather than arriving at specific recommendations for sample sizes, a method for calculating 95% confidence intervals on Benn diagrams is presented based on the work of Ringrose and Benn [28–30] and on what has been proposed by Weaver and Steele [31, 32] for assessing mortality profiles in faunal assemblages also ternary diagrams. With confidence intervals, the interaction between the underlying orientation tendencies and sample size are exposed. These results confirm that the size of the confidence interval for a given sample size depends very much on the structure of the data or, in other words, the type of post-depositional disturbance that may have occurred. Confidence intervals also allow for a better assessment of whether and what kinds of post-depositional disturbances may have affected the deposit by showing potential overlap between the uncertainty in the observed data and the convex hulls of sets of points known already to represent various kinds of processes [5].

Second, another difficulty in orientations analysis is in knowing just how similar or different assemblages are in their three dimensional orientation structure (i.e. after having computed eigenvalues and plotting these on a Benn diagram) [28]. Though there are tests for comparing bearing angles (e.g. Rayleigh test) or plunge angles (e.g. unpaired t-test), to my knowledge there exists no recommended test for comparing the two together. The above mentioned confidence intervals on the Benn diagrams do help give some indication of the degree to which assemblages may differ [27, 29, 33], and here an additional option of applying a permutations analysis to the Euclidean distance between the Benn indices (elongation and isotropy) is presented to address this problem.

Third, there is a lack of tools for exploring spatial variability in orientations. If there are reasons to segment an assemblage into spatially distinct sets, then the above techniques allow them to be compared. However, when there are no a priori reasons to spatially segment an assemblage, a method is needed to examine whether there is, nevertheless, spatial patterning in the data. To address this, a method for calculating and plotting nearest neighbor samples and, in effect, moving averages across an assemblage from a particular stratigraphic unit is presented. This method allows spatial patterning across an assemblage to be visualized in Benn space. When patterns are detected, the assemblage can then be spatially segmented and tested following the above mentioned techniques.

Additionally, to further advance the analysis of artifact orientations, all of the software used in this paper to make the standard [1] and the newly presented statistical and graphical techniques are available in the Supplemental Information. This code, written in R [34], addresses an additional shortcoming, namely easy access to tools needed to do this type of research.

## Materials

Three data sets are used here: a simulated one, a previously published one, and a recently excavated one. The simulated data set consists of varying proportions of a perfectly planar assemblage, meaning objects laying on a surface and oriented in all directions on this surface, and a nearly perfectly linear assemblage, meaning objects all oriented in exactly the same direction (see Lenoble and Bertran [5] for illustration of these terms). To avoid having these assemblages fixed to the base line of the Benn diagram (see Methods below) and to make these simulated assemblages more like archaeological ones, a uniformly random dispersion from horizontal of up to 10 degrees has been introduced into these data (lifting them slightly towards the isotropic pole of the Benn diagram). Because a perfectly linear assemblage would not have variability that could be meaningfully resampled, the linear assemblages are drawn from horizontal angles uniformly, randomly distributed from 160 to 200 degrees (20 degrees to either side of grid south) and with the same vertical dispersion of 10 degrees. From these two assemblage types, mixed assemblages are created in varying proportions of 0%, 20%, 40%, 60% and 80% linearly oriented objects (the rest being drawn from the planar assemblage) and with varying assemblage sizes in increments of 20 from 30 to 150. The R code to create, analyze and present these simulated assemblages is provided in the Supplemental Information.

To help interpret the results presented here, the comparative orientations data from natural slope deposits published by Lenoble and Bertran [5, 35] were digitized using the open source program WinPlotDigitizer Version 3.8. These data for debris flow, for runoff on steep and shallow slopes, and for solifluction are used here to provide some indication of the range of Benn values for these formation processes. This in turn helps to better understand whether a particular 95% confidence interval for an assemblage includes the possibility of more than one interpretation for the formation of the deposit. Note that for solifluction, one extreme outlier in the published data set is excluded here because it falls so close to the planar position that this must represent a type of solifluction quite distinct from the remaining data points as to make interpretation less meaningful (note that [36] also drops this point from the solifluction comparative data set).

The excavated data set comes from recent excavations, led by Alain Turq, at the site of La Ferrassie (Table 1) [37, 38]. The 3m La Ferrassie stratigraphic sequence consists of multiple Middle and Upper Paleolithic levels with bone and stone artifacts exposed over several square meters. As summarized in [37, 38], the sediments are fluvially deposited sands and gravels overlain by slope deposits. The transition between these two depositional regimes occurs in Layer 3, and Layer 2 shows evidence of deposition and subsequent alteration under a cold climate regime. In excavating these deposits, all bones and stones larger than 2.5 cm were piece provenienced with a total station (typically with 5 second precision). All such elongated objects were recorded with two points, one at each end of the long axis (see [1]). What constituted elongated was not numerically defined. Rather excavators were initially instructed on examples of elongated objects and the reasons for recording and analyzing two points. The site supervisor helped with initial determinations and was always available to provide some control. However, in our experience, excavators quickly develop a sense of which objects merit two points. For a subset of the stone artifacts (the complete flakes and tools) from the Middle Paleolithic layers, the elongation can be calculated from the length and width measurements taken during subsequent stone tool analysis. Length here is recorded from the point of percussion to the furthest point, and width is recorded at the midpoint of this line and perpendicular to it. The median length of the long axis of all objects is calculated based on the Euclidean distance between the two total station coordinates for each object. Both of these measures, elongation and length, can be used to filter from analysis artifacts that are perhaps not elongated enough to have had a clear principal axis or artifacts that are too small when there are some doubts about the quality of the recorded data or when, for instance, examining the effect of post-depositional processes on different size or shape classes. Here these values are reported, but they were not used to filter the data set.

**Table 1:** La Ferrassie two-shot sample characteristics by layer. Elongation mean and standard deviation are calculated on a sub-sample of lithics with length and width measurements made with calipers (see text for how these measurements were made). Median length is calculated based on the two total station coordinates for the object.

	Bones	Lithics	Length (Median)	Elongation (n)	Elongation (Mean)	Elongation (SD)
7B	65	22	0.039			
7A	460	159	0.042			
6	25	53	0.046			
5B	195	36	0.049	32	2.35	0.66
5A	171	15	0.055	8	1.51	0.88
4	339	37	0.058	29	2.29	0.84
3	242	33	0.051	26	2.22	0.87
2	189	14	0.058	13	2.49	0.63
1	23	9	0.063	9	1.83	0.94

The La Ferrassie data summarized in Table 1 and presented below are provided in the Supplementary Information as an R data object.

## Methods

The orientation of an object’s long (a-) axis line can be described by the geological terms bearing or trend and plunge [39, 40]. Bearing is the horizontal angle of the long axis line relative to some geographic or arbitrary north, and the plunge is the vertical angle of the line relative to the horizontal plane. An object with a plunge angle of 0 is lying level on a horizontal plane, and an object with a plunge angle of 90 is perpendicular to the horizontal or straight up and down. Normally bearing angles range from 0 to <360 degrees. An object with a bearing of 0 degrees is pointed due north (positive y-axis) in the site grid, and an object with a bearing of 90 is pointed due east (positive x-axis) in the site grid. However, without the added information coming from the plunge angle, an object with a bearing of 0 is indistinguishable from its complement (180 degrees). By considering also the plunge angle, an object can then be said to be plunging towards the north (bearing is 0 degrees) versus to the south (bearing 180 degrees). In what is presented here, bearing angles vary across 360 degrees and are visualized as plunging in the bearing direction using Schmidt lower hemisphere diagrams with a superimposed Rose diagram (see [1]). So for clarity, here the term orientation refers to the general alignment of an object in three dimensional space and the terms bearing and plunge, as just defined, are used to describe the respective horizontal and vertical components as necessary. Note that bearing is periodic or circular and as such requires special statistical treatment whereas plunge is not circular and can be treated with ordinary descriptive statistics. Here, however, the two are treated together as a vector describing the orientation of the object.

Some descriptive statistics and visual methods for treating orientation data are presented in McPherron [1] and citations within. One of these methods, specifically the use of Benn ratios, is the basis of the new techniques presented here. Benn [4] showed that two ratios, elongation and isotropy, calculated from computed eigenvalues [41] and plotted on a kind of modified ternary diagram are useful for summarizing assemblage orientations. Eigenvalues represent the degree of clustering around three mutually orthogonal eigenvectors. They sum to 1, and the first eigenvalue is the largest, followed by the second and then the third. Thus how this sum is distributed across the three eigenvalues says something about the orientations. The first eigenvalue represents the maximum clustering in the data. A high value for this eigenvalue and a low value for the other two indicates linear orientations (all objects pointed generally in the same direction). When the first two eigenvalues have roughly equal values and the third a lower value, then the orientations are planar (objects randomly oriented on a plane). When all three eigenvalues are roughly equal then there is no preferred orientation, and the data are considered to be isotropic (randomly oriented in three dimensions). The elongation and isotropy ratios capture this patterning. The elongation ratio is computed as 1 minus the ratio of the second eigenvalue to the first. Thus when the first eigenvalue is large in comparison to the second, their inverse ratio becomes smaller and the elongation ratio approaches 1. When the two values are roughly equal, the inverse ratio approaches 1 and the elongation ratio then approaches 0. The isotropy ratio then captures the relationship between the third eigenvalue and the first (again expressed as ratio of the two values but this time without subtracting from 1). Now when the first eigenvalue is large in comparison to the third, the inverse ratio of the two approaches zero, and to the contrary when they are roughly equal, the isotropy ratio approaches 1. Thus a perfectly isotropic assemblage will have an elongation ratio of 0 and an isotropy ratio of 1. A perfectly linear assemblage will have an elongation ratio of 1 and an isotropy ratio of 0. When both ratios are 0, the assemblage is planar. This approach has the advantage of simultaneously considering the bearing and the plunge to describe orientations, and it is well suited to the kind of a-axis orientation data that the total station recording method provides.

The code to implement the methods described below is written in R [34] by the author. This code makes use of the CircStats [42], colorspace [43–45], KernSmooth [46], fields [47], spatial [48], and tiff [49] packages. This document (included in the Supplemental Information as well in rmarkdown format) was prepared using the dplyr [50], reshape [51], knitr [52–54], xtable [55] and knitcitations [56] packages.

To calculate confidence intervals in Benn space (elongation vs. isotropy indices), the Benn space is divided into 100 intervals (representing.01 increments of each index) or a square matrix of 10,000 individual cells. The original assemblage is then resampled with replacement repeatedly, and with each resampled assemblage Benn indices are calculated and totaled by the respective cell. In the end, the 100 x 100 matrix is examined to determine what count value represents the threshold at the appropriate probability value (typically.95). At this threshold, the sum of the cells with counts above this value account for, for instance, 95% of the resampled assemblages, and the remaining cells lie outside the 95% confidence interval. The contourLines function (grDevices) is then used to calculate the appropriate contour lines through this matrix along the two Benn indices, and the results are plotted. The choice of a 100 x 100 grid is arbitrary and intended only to give a satisfactory visual result. Resampling is repeated 10,000 times (cf. [28]). Again, this is an arbitrary decision intended to trade computational time against visual results. Resampling fewer times simply results in rougher contour lines often also with small, isolated pockets of probability at the edges of the central 95% contour.

While the method proposed here is based on one presented by Ringrose and Benn [28–30], it differs from theirs in the following way. Ringrose and Benn noted that when there are eigenvalues of similar magnitude (e.g. the first two eigenvalues for planar fabrics and all three for isotropic fabrics), resampling can result in these eigenvalues swapping places such that, for instance, the first eigenvector becomes the second and vice versa. What this does, in turn, is reduce the amount of variability that can occur in the resampling as eigenvectors and their associated eigenvalues that would have otherwise moved further apart are swapped thereby decreasing their apparent differences. In addition, when samples plot close to the left edge of the Benn diagram, e.g. when the fabric is planar and the first two eigenvalues are nearly equal in magnitude, resampling will less frequently result in eigenvalues of more similar magnitude and more frequently result in less similar eigenvalues. This then will impact the shape of the 95% confidence interval by making the resampled points drift to the right of the original sample point (see [28] for a complete explanation). While noting that drift is to some extent unavoidable in the eigenvalue approach, Ringrose and Benn [28] propose retaining the original order of the eigenvalues (thereby violating the rule that they decrease in magnitude) by inspecting the eigenvectors to note when they have been swapped, re-ordering the eigenvectors and associated eigenvalues to reflect the original order of the eigenvectors, and expanding the simple one panel Benn diagram into a six panel diagram to account for the new possible arrangements of the eigenvalues. In what is presented here, the eigenvalues are allowed to swap positions, and panels are not added to the Benn diagram. This decision was made for three reasons. First, an informal assessment of the impact of eigenvector swapping on the 95% confidence intervals showed that practically speaking its impact on the location of the confidence interval on the main Benn diagram was relatively minor. Not adding additional panels means that the resampled assemblage often plots to the left of the 95% confidence interval rather than at its center, but, second, the proposed multiple panel diagram solution has not been widely accepted (but see [33] and [27]) meaing that, third, nearly all of the comparative data used to interpret the plots uses the standard Benn diagram. Thus while the Ringrose and Benn proposal is preferable from a statistical perspective, it is not practical at this time and, importantly, not applying it does not appear to change the interpretation of deposit formation based on fabric orientation. Nevertheless, the code to compute the resampled eigenvalues following Ringrose and Benn is included in the attached files.

To assess the likelihood that two assemblages come from the same population (i.e. the null hypothesis that they have the same orientations cannot be rejected), a permutations test is applied. To do this, first, the Euclidean distance between the two assemblages in Benn space is calculated (i.e. the distance between where they plot using the elongation and isotropy indices). Next, the two assemblages are combined, and two new assemblages are drawn randomly from this combined assemblage without replacement and with sample sizes equal to the original two assemblages respectively. The Euclidean distance between the Benn indices of these resampled assemblages is calculated. Finally, this step is repeated a set number of times to create a distribution of expected distances if the two assemblages represent a random partitioning of a single population into two assemblage groups. Once this is done, the originally calculated actual distance between the two assemblages is compared to the distribution of expected values to calculate its probability. This is a one-sided probability because we are interested in instances where the distance is more (not less) than we would expect based on resampling.

To view spatial variation in object orientations, Benn indices are calculated for every object on a sample made of neighboring objects. What counts as neighboring is arbitrary and could be based on distance or sample size. The two are typically directly related, but here the nearest 40 objects, rather than a fixed distance, are used. This value was chosen arbitrarily (and can be varied) to have enough objects in each sample to reasonably compute Benn indices (while confidence intervals could be computed for each of these samples, it is not obvious how to visualize this). One drawback to this approach is that, in some cases, depending on how the densities of objects varies spatially, objects from fairly far away (or outside what one would consider to be the local area of interest for that object) might get included in the sample. To protect against this, a maximum distance threshold is included in the calculation as an option. This option is not applied here because in general the spatial arrangement of the objects is suitable and because it can result in samples of varying sizes. Next, the space within the Benn diagram is given a color coding that varies from green to red from the planar to the linear poles respectively (changes in the elongation index) and varies in saturation (amount of white mixed into the color) based on the distance from the isotropic pole (isotropy index). Each local assemblage is plotted on the Benn diagram in black but the corresponding color is used to plot the object on a plan view of the entire assemblage. In this way, the Benn diagram then becomes both a key to reading the spatial plot and itself a representation of variability within the assemblage not unlike the 95% confidence intervals but in this case spatial structured. A large variation in the distribution of points on the Benn diagram will then suggest that spatial differences exist within the assemblage, and these can be further examined with reference to the color coded plan views. This method is purely exploratory and offers no statistical tests of significance or descriptive statistics of the spatial patterning. However, this can be achieved easily by then dividing the assemblage into spatially defined groups based on inspecting the results visually and by then calculating standard orientation statistics on these groups (including the above described permutations test for differences between groups).

## Results

**Fig 1:**
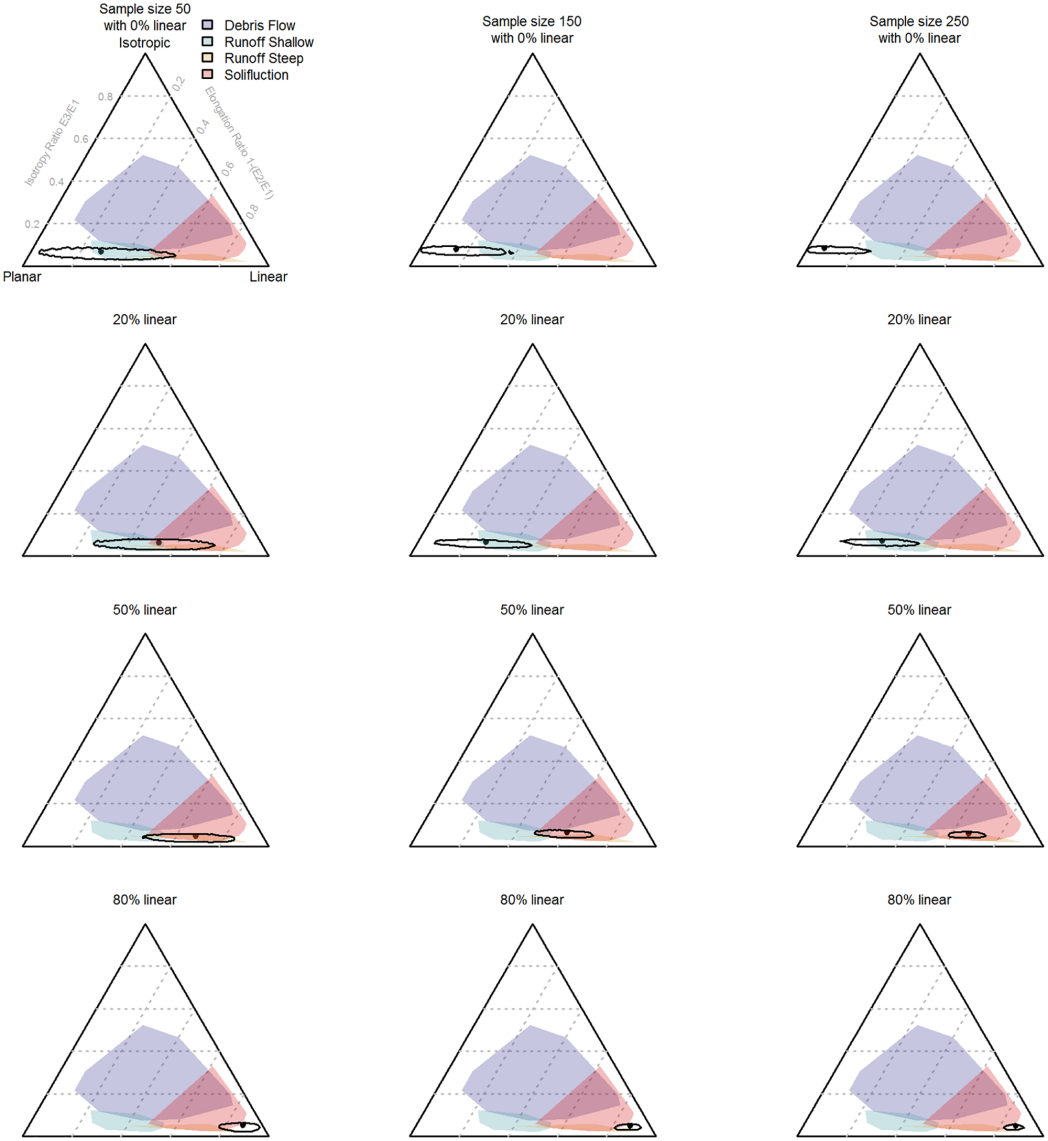
Confidence intervals on simulated data showing the effect of sample size with varying proportions of planar and linear data. The black dot in each case shows the actual calculated value for that assemblage. Each column of figures has the same sample size. Each row of figures has the same proportional mix of planar and linear samples. E1, E2 and E3 are the first, second and third eigenvalues respectively. Comparative deposition data from [5].

**Fig 2:**
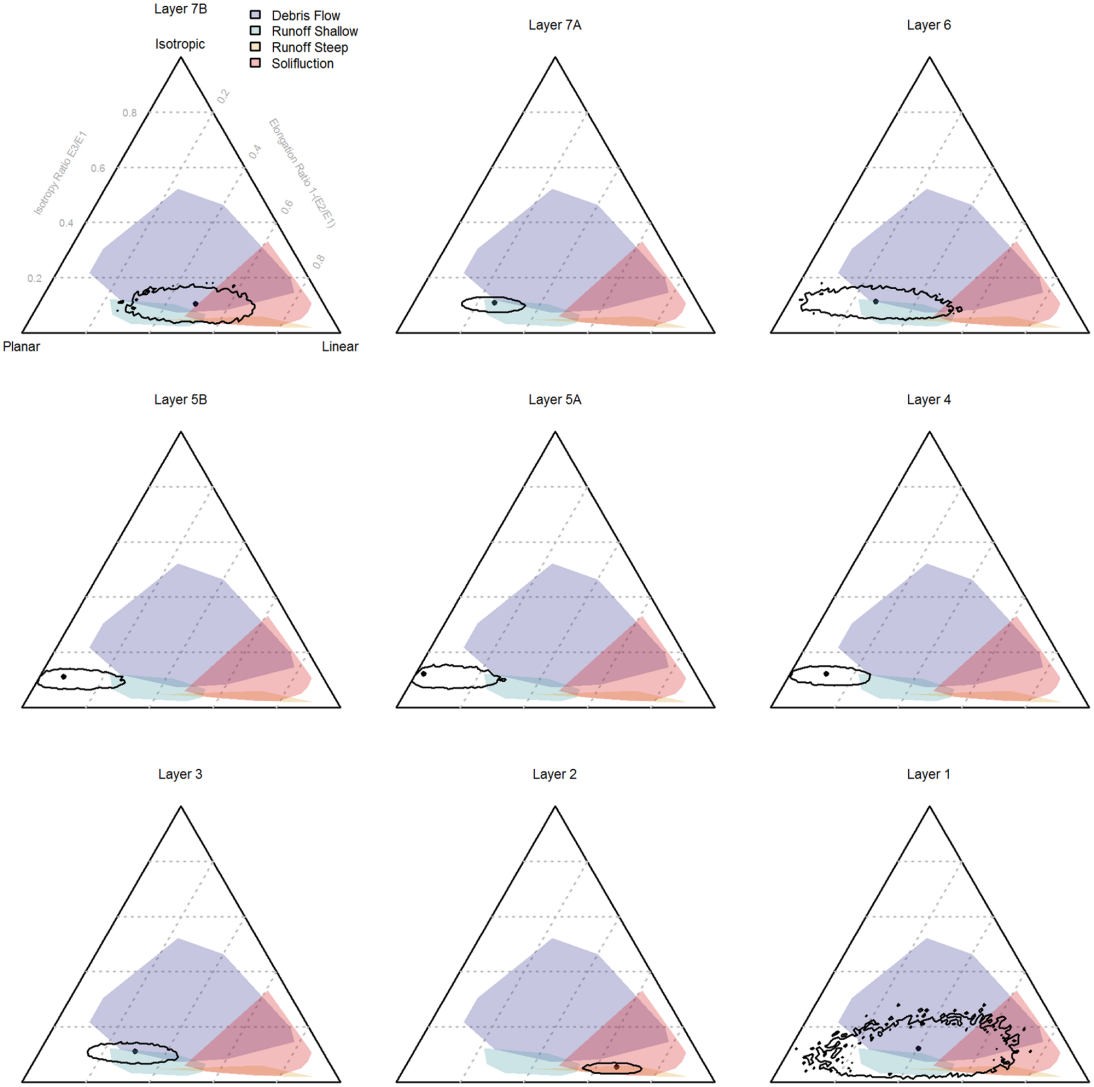
Benn diagrams for each La Ferrassie layers. The dot indicates the calculated values for that layer. The confidence intervals represent a probability of 0.95 based on 10000 times resampling.

**Fig 3:**
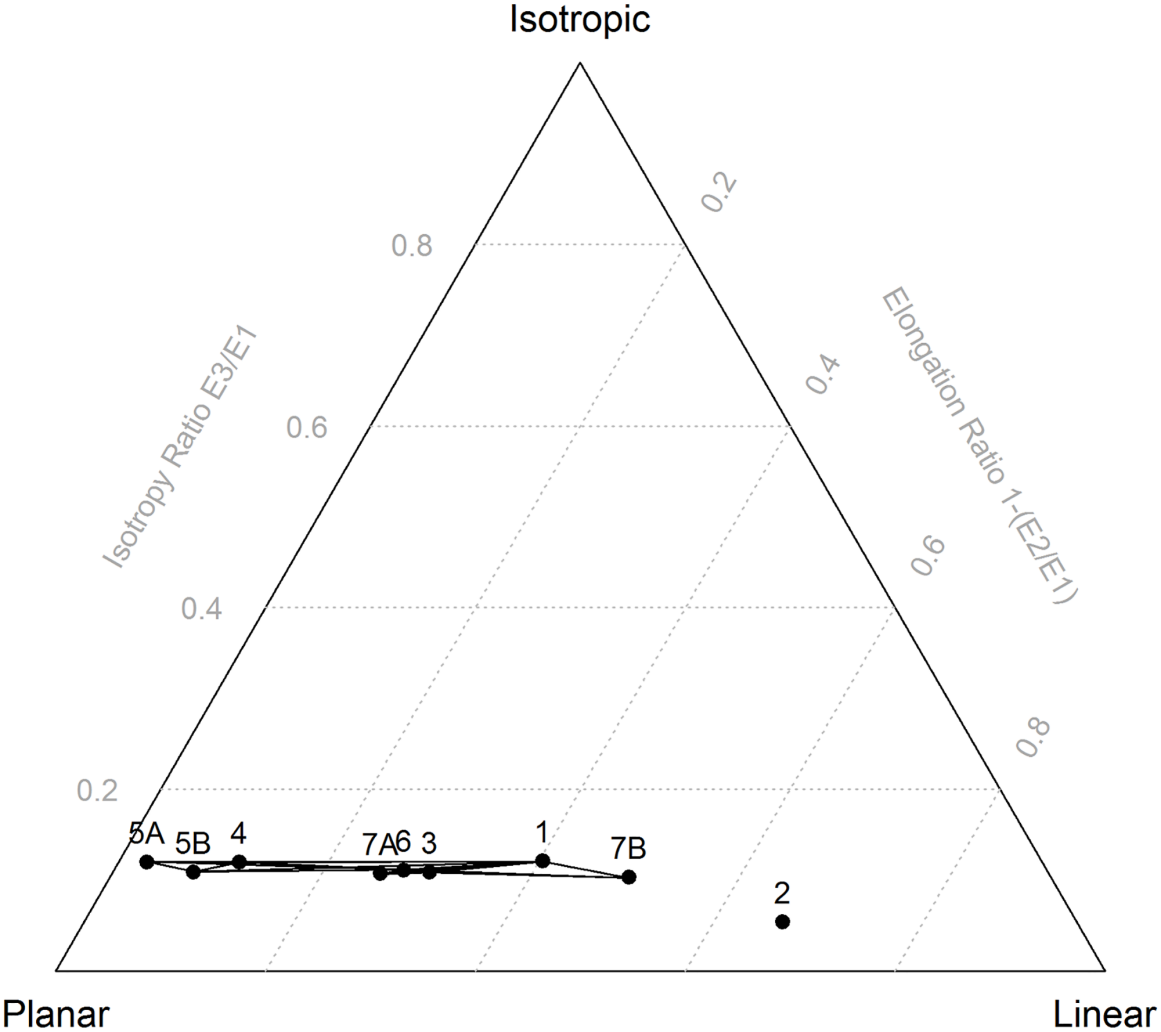
Benn diagram for all La Ferrassie layers. Connecting lines indicate layers that do not differ statistically at the p = 0.05 level based on a permutations test with 10000 times resampling (see Table 2).

**Fig 4:**
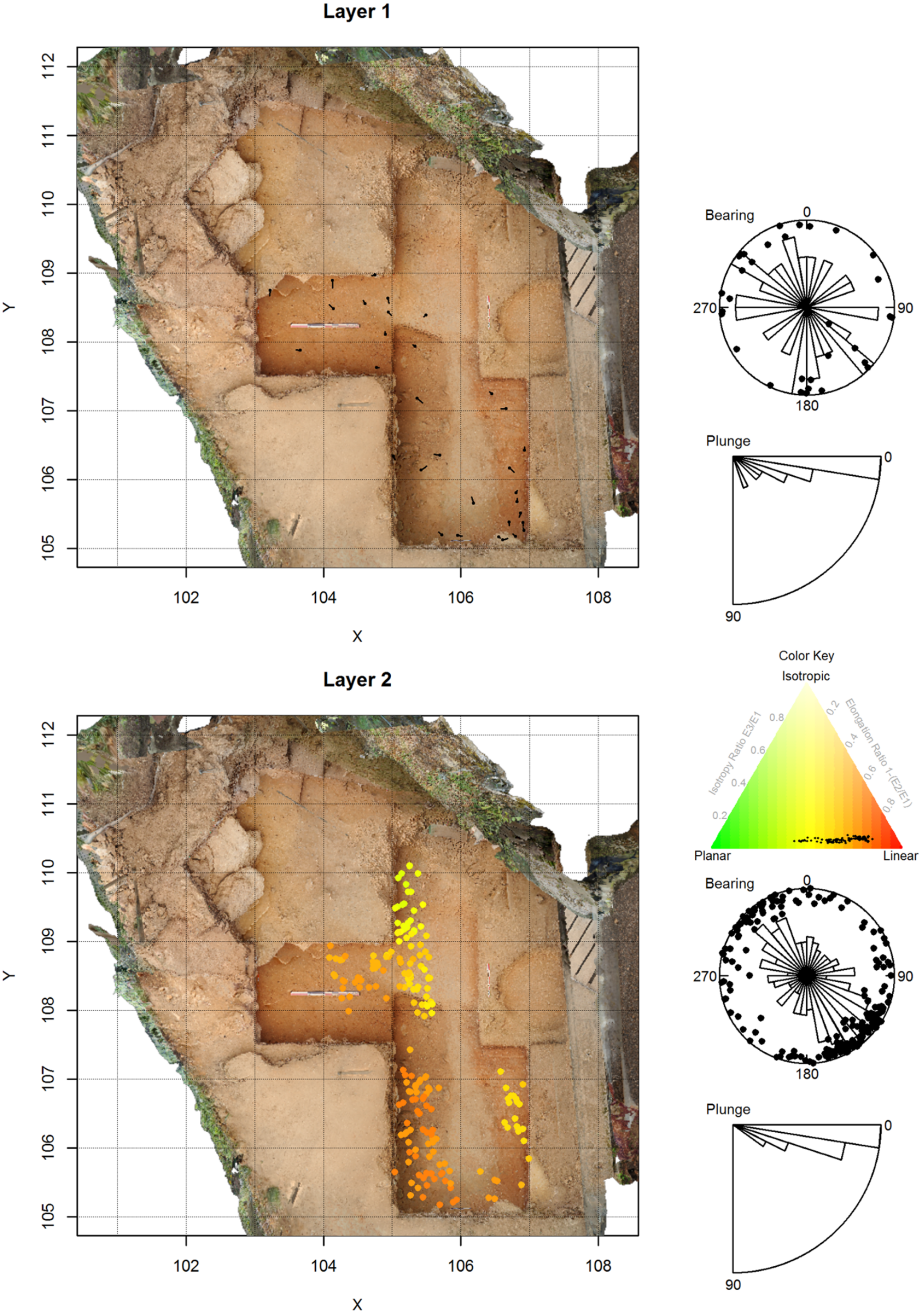
Artifact orientations for La Ferrassie Layers 1 and 2. The plan view shows all two-shot bone and stone artifacts. The underlying image is a georeference orthophoto extracted from a structure from motion model. For Layer 2 the plan view points are color-coded using Benn statistics computed on the 40 closest artifacts. The color key is shown in the Benn diagram where these same points are also plotted as black dots. Each figure also includes a Schmidt lower hemisphere plot summarizing bearing and plunge angles with a superimposed Rose diagram summarizing only the bearing angle distributions [1]. Below this is a circular histogram of plunge angles. Though the sample size in Layer 1 is not large enough for spatial color coding, the layer is included here for completeness of the presentation of La Ferrassie artifact orientations.

**Fig 5:**
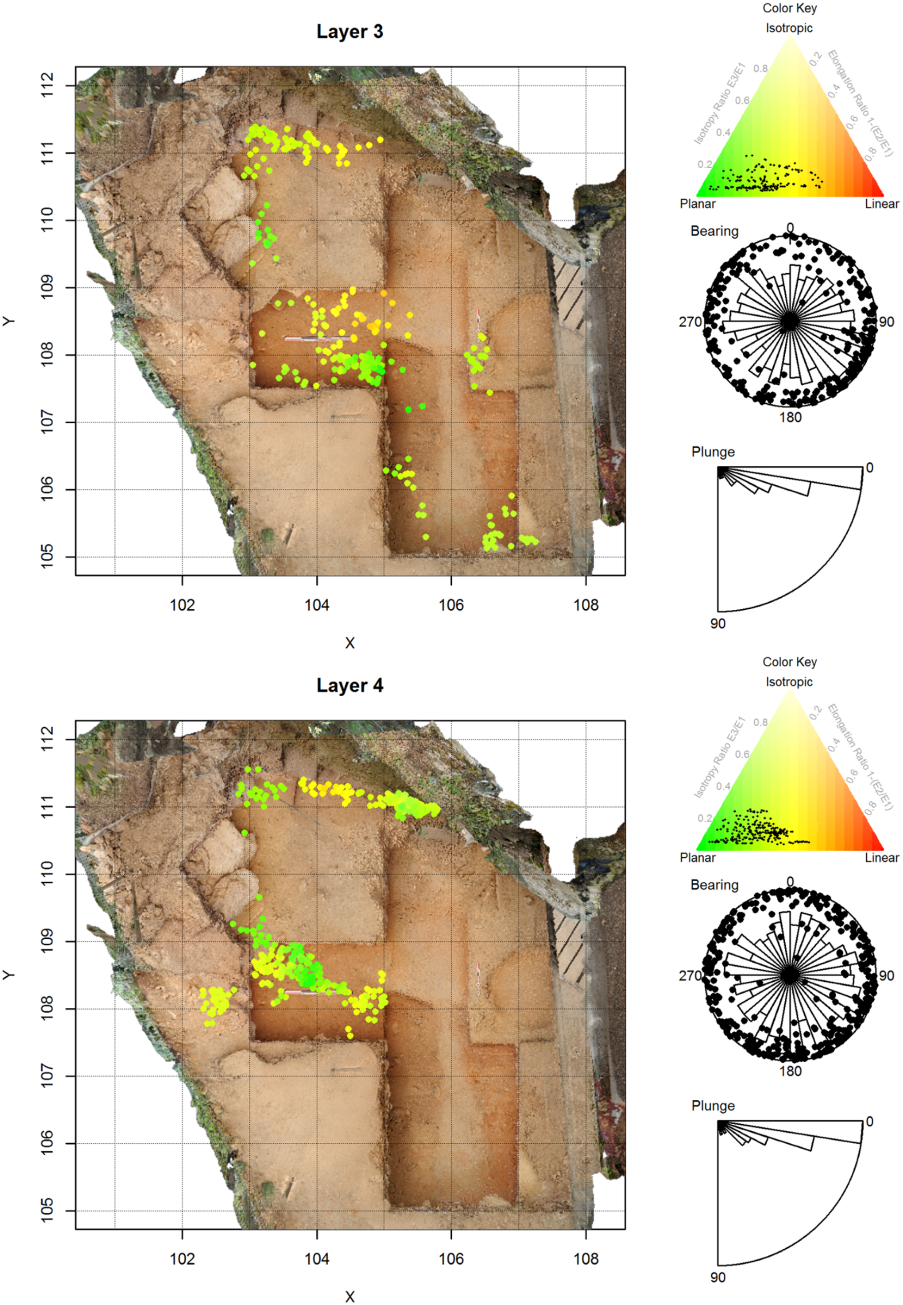
Artifact orientations for La Ferrassie Layers 3 and 4. See Fig 4 for an explanation of the figure elements.

**Fig 6:**
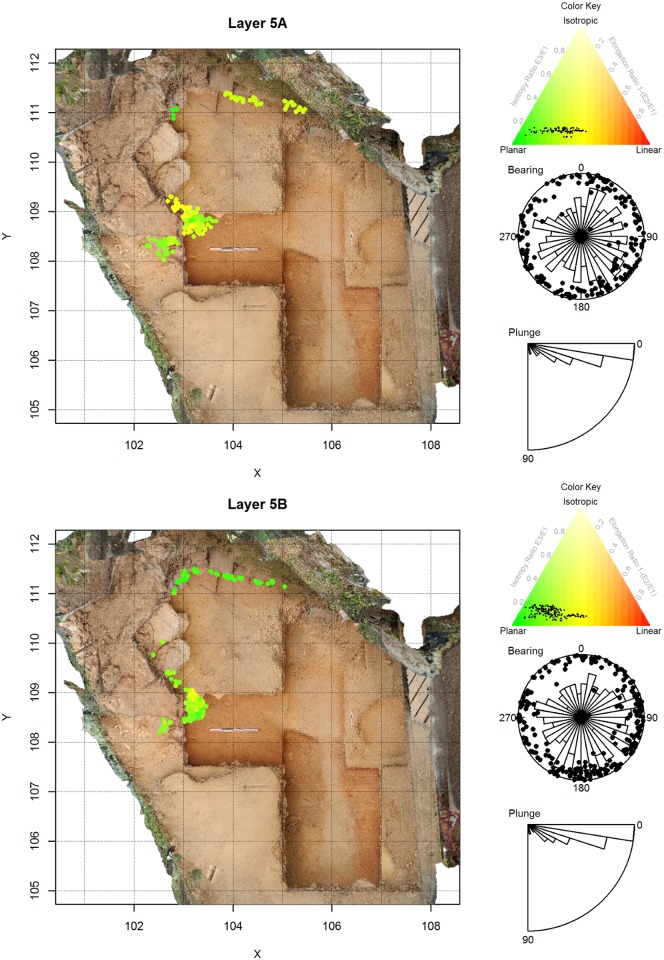
Artifact orientations for La Ferrassie Layers 5A and 5B. See Fig 4 for an explanation of the figure elements.

**Fig 7:**
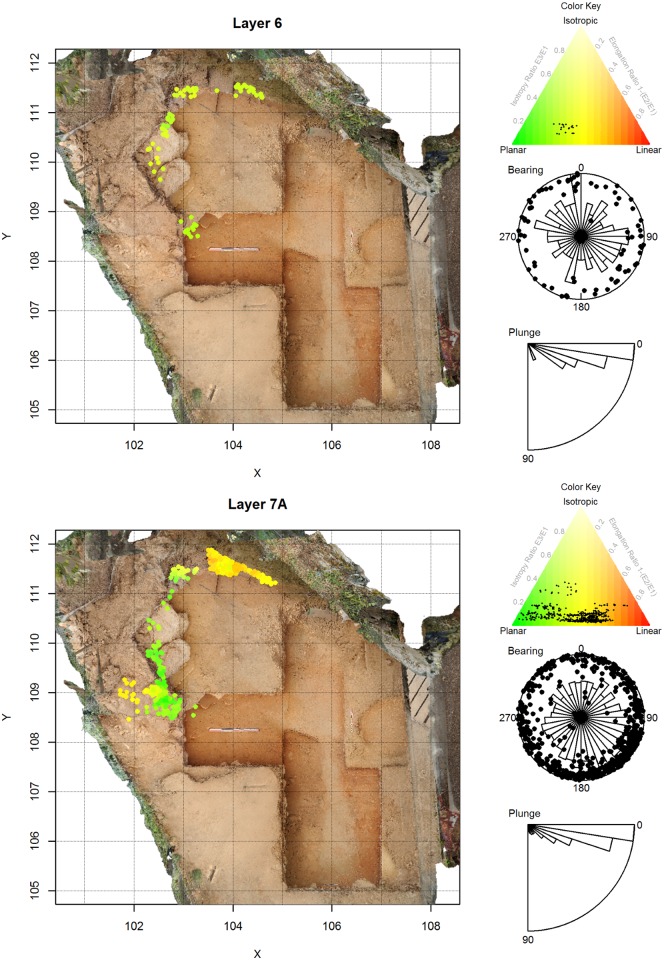
Artifact orientations for La Ferrassie Layers 6 and 7A. See Fig 4 for an explanation of the figure elements.

**Fig 8:**
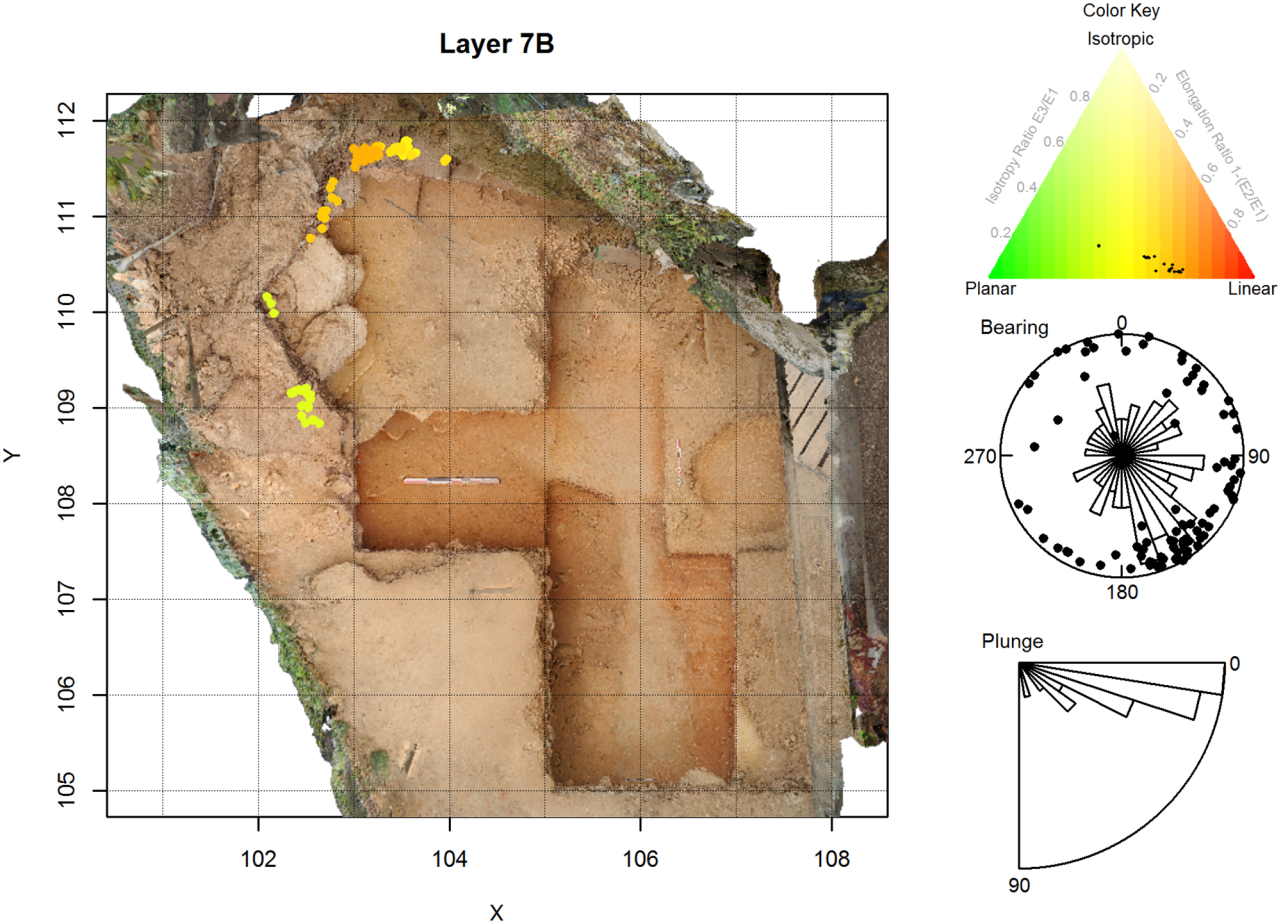
Artifact orientations for La Ferrassie Layers 7B. See Fig 4 for an explanation of the figure elements.

**Fig 9:**
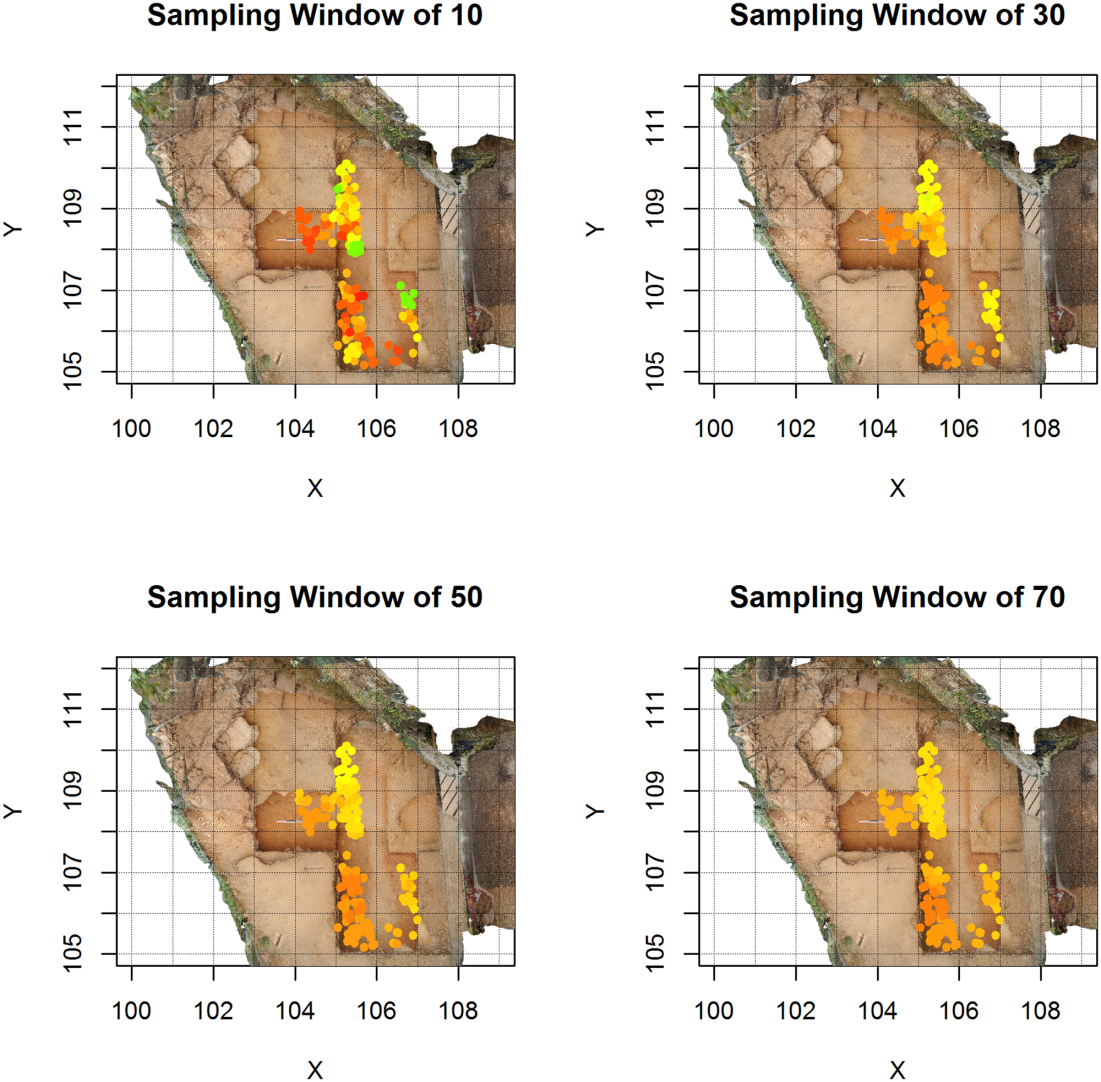
Artifact orientations for La Ferrassie Layer 2 with varying sampling windows (see also Fig 2 for the color key and for a sampling window of 40). With a small sampling window more small scale variation is apparent but it is less clear whether this is meaningful variability or sampling bias. With a large sampling window, the spatial variability in this layer is less apparent as the color of each individual point approaches the assemblage average.

The effect of sample size and of varying proportions of planar and linear objects on the 95% confidence intervals of the Benn ratios and on the range of possible interpretations is demonstrated in Fig 1. In Fig 2, the Benn ratios and 95% confidence intervals are shown for all the main layers from the La Ferrassie excavations. Fig 3 and Table 2 show the results of pair-wise permutations test between the Benn ratios for each layer combination. Finally, Figs 4–8 show how the Benn ratios vary spatial within each layer, and Fig 9 shows how changing the sampling size window impacts the results for La Ferrassie Layer 2.

**Table 2:** Probabilities resulting from permutation test results of layer pair comparison. Results based on 10000 times resampling. Probabilities > 0.05 are plotted with connecting lines in Fig 3.

	1	2	3	4	5A	5B	6	7A	7B
1		0.01	0.60	0.14	0.05	0.09	0.51	0.39	0.64
2			0.00	0.00	0.00	0.00	0.00	0.00	0.03
3				0.03	0.01	0.02	0.91	0.59	0.11
4					0.32	0.71	0.19	0.05	0.00
5A						0.56	0.06	0.01	0.00
5B							0.12	0.03	0.00
6								0.95	0.13
7A									0.03
7B									

## Discussion

The results of the resampling of simulated assemblages varying in their proportion of linear and planar objects demonstrates that the size of the confidence intervals vary in two predictable ways. First, as the sample size increases, the size of the confidence interval decreases (reading across each row of Fig 1). Second, the size of the confidence interval depends on the structure of the underlying sample. If the sample is more linear, then the confidence interval will be smaller (reading down each column of Fig 1). Conversely, planar assemblages have larger confidence intervals. What this means practically is that, for instance, by the time 50 objects are measured, if the sample shows a tendency towards linear orientations, then one can be fairly confident that indeed the sample comes from an assemblage of linearly aligned objects. However, conversely, if the assemblage plots more towards the planar pole on the Benn diagram, then there will still be a large uncertainty in the result. Assemblages that initially appear to be good (i.e. close the planar pole) may in fact show patterns more indicative of post depositional disturbances after a larger sample is collected and vice versa. Though it was not modeled here, increased isotropy would behave like planar assemblages in that low sample sizes will have larger uncertainties than either linear or planar assemblages of the same size. In other words, with more variability in the sample, there is less confidence in whether this variability is a function of sampling bias or representative of the true underlying pattern. As a result, there can be no one recommendation for a minimum sample size for artifact orientations. The best practice recommendation is to collect orientation data until the size of the confidence interval is sufficiently small that the range of possible interpretations for the formation of that deposit is reduced. If it is important to test whether one assemblage is distinct from another (see below) a large sample may be required, and in any case, orientations should be reported with confidence intervals.

Note that when orientation data are collected with a total station and data collector, the code provided here makes it practical to process the data on a daily basis and adjust excavation strategies accordingly. So, for instance, in deposits that are low in artifact densities, a decision can be made to record small natural clasts, often times not normally recorded during excavation, to increase sample sizes. While perhaps in some instances natural clasts and artifacts may have different orientations and while this is in itself a potentially interesting line of investigation which can be tested using the permutations technique, at least the combined sample could give a greater confidence on the formation of a particular deposit. Once this is achieved, the excavation strategy can then be modified to return to the more standard approach of not documenting in detail small natural clasts.

The results for the La Ferrassie layers indicate that with the 95% confidence intervals only Layers 4 and 5 plot close to the planar portion of the Benn diagram with very little overlap (i.e. a low probability) with comparative data for depositional processes such as solifluction, run-off or debris flow. Especially Layer 1 but also Layer 6 have low sample sizes and this is reflected by their large confidence intervals. In both instances, it is not clear from the sample we have whether the objects in this layer have moved after deposition or not. Layer 1 could represent almost any kind of deposit, based on the orientations only, whereas Layer 6 is more likely either undisturbed or disturbed somewhat by objects shifting downslope. Layers 3 and 7 also show some post-deposition alteration consistent with downslope movement. The position of Layer 2 is consistent with either solifluction or movement on a steep slope. Note, in keeping with the results of the simulated data sets, the confidence interval is much smaller than seen for Layers 5a and 5b despite similar sample sizes.

The results presented here also show that permutations test are an effective way to compare the orientations of two assemblages in three dimensions. This is useful because Benn diagrams, which summarize both the bearing and plunge information in an assemblage of oriented objects, have been shown to be such a useful tool for interpreting site formation processes. Here the La Ferrassie data set is used to illustrate the method. The results follow the 95% confidence interval results and show, for instance, that indeed Layer 2 is unlike any of the other layers and that Layers 4, 5A and 5B are quite similar to each other but different from the underlying Layer 3. However, in addition to comparing whole layers in a site like La Ferrassie, for instance, the method gives a useful tool for comparing sub-assemblages. With permutations testing, the likelihood that natural stones and artifacts or that bones and stones or that small and large objects have the same orientations can be assessed. Permutations testing returns a specific probability (based on randomized, repeated resampling) that can be used for null hypothesis testing. However, it can also be used simply as a relative measure of the similarity of two assemblages.

Finally, based on its application to the La Ferrassie data set, the graphical and statistical technique applied here to presenting spatial variation in object orientations appears to be effective. For instance, though the above results showed that Layer 2 had been post-depositionally altered, the spatial results show that consistent differences are clearly visible across the layer with one portion showing more heavily aligned artifacts than the other. In fact, this difference corresponds to a channel like feature visible during excavations and in the remaining profiles and that likely represents a solifluction lobe. In other layers of La Ferrassie, especially Layers 7A and 7B, differences can be seen in the orientations of objects in the grid north section of the site. This portion of the excavation area is near or immediately adjacent to the cliff face and likely represents some type of “wall effect” common in cave sites. So while the assemblage averages with 95% confidence intervals for Layer 7 indicate some post-depositional alteration, the spatial data reveal a more complex picture with parts of the layer looking intact while other parts have clearly suffered some alterations. Thus this method allows for a more nuanced view of site formation processes, especially when combined with permutations testing to help quantify the magnitude of observed differences.

One important issue with this approach is the selection of the size of the sample window used to calculate the Benn statistics for each object. On the one hand, small sample sizes may be more sensitive to small scale variations in object orientations, and this could be important for interpreting the formation of a deposit; however, they will also be subject to random variations related to biased sampling (see above). Further, the resampling and confidence intervals approach implimented here demonstrates that the sensitivity of the size of the sampling window to random variation is dependent on the structure of the orientations. On the other hand, a large sampling window may obscure important patterning. Thus there is no simple solution. The best practice recommendation in this instance is to try windows of different scales and assess the results in each case (Fig 9). This approach is similar to what is recommended in other types of spatial analysis, and it may in fact be useful to apply some additional spatial statistics to quantitatively assess whether the resulting spatial patterns at a particular scale or sample size depart from expectations under a random model.

## Conclusion

The goal of this paper has been to provide some possible solutions to methodological issues that exist with the analysis of object orientations. The issues addressed here are what sample size is required for secure interpretation of site formation processes, how to make pair-wise probability based comparisons of assemblage orientations in three dimensions, and how to explore and represent spatial variability in orientations within an assemblage. The methods presented here are proposals and consist respectively of the use of confidence intervals to assess sample sizes, permutation testing to assess assemblage differences, and moving sampling windows to visualize spatial variability. As for the latter method, the application of spatial statistics to the observed spatial patterns is an obvious next step that will help bring additional statistical support for the interpretations of observed patterning.

R code to implement each of these methods as well as the complete example data set from La Ferrassie are included here with these proposals. It is clear that as researchers increasingly rely on results coming from program code, a proper evaluation of the results requires not just the data but also the code [57]. Thus, here the general routines needed to do the orientations analysis are published, but also included is the document of mixed text and code used to build this manuscript with all of its tables and figures. It is hoped that researchers looking at the topics discussed here will find this useful and also that this will contribute to a growing trend in open science publication.

## Supporting information

S1 File R Markdown file used to create this document. (RMD)

S2 File Le Ferrassie artifact orientation data set. (RDS)

S3 File Lenoble and Bertran (2004) comparative data set. (RDS)

S4 File R source code to implement the methods presented here. (R)

S5 File Aerial image of La Ferrassie. (TIF)

S6 File Georeference file for aerial image of La Ferrassie. (TFW)

S7 File Citations included within this manuscript. (BIB)
