# Supplementary figures and images for: Additional statistical and graphical methods for analyzing site formation processes using artifact orientations
Source: PLoS One. 2018 Jan 2;13(1):e0190195. doi: 10.1371/journal.pone.0190195 (PMC5749765; doi:10.1371/journal.pone.0190195)

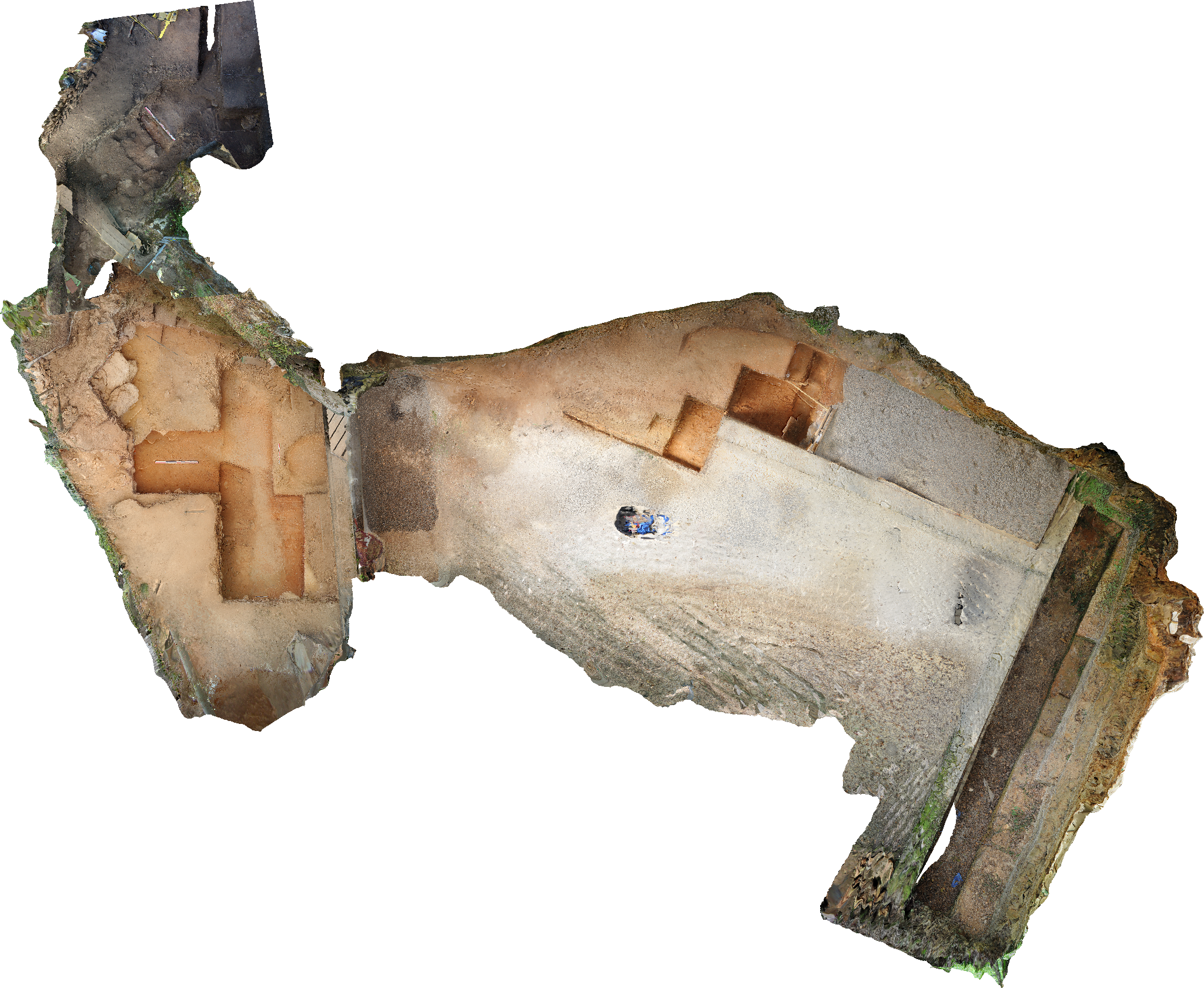

Supplement: S5 File — (TIF) [file pone.0190195.s005.tif]
